# Links between mothers’ ACEs, their psychopathology and parenting, and their children’s behavior problems–A mediation model

**DOI:** 10.3389/fpsyt.2022.1064915

**Published:** 2022-12-22

**Authors:** Keren Hanetz-Gamliel, Daphna G. Dollberg

**Affiliations:** School of Behavioral Sciences, The Academic College of Tel Aviv-Yaffo, Tel Aviv-Yafo, Israel

**Keywords:** mother’s ACEs, mother’s psychopathology, parenting behavior, child’s internalizing behavior, child’s externalizing behavior

## Abstract

**Introduction:**

Children of mothers with a history of adverse childhoods are at greater risk of behavior problems. However, the mechanisms through which a mother’s early adverse experiences (ACEs) are transmitted to her children need further study. Our goal was to examine a conceptual mediational model linking mothers’ ACEs, maternal psychopathology symptoms, and parenting behaviors with children’s internalizing and externalizing behaviors sequentially.

**Methods:**

A sample of 153 Israeli mothers of children ages 3-12 (52% girls) participated in the study, and most of the mothers (94.7%) were cohabiting with a spouse. Mothers completed online questionnaires about their early adverse experiences, psychopathology symptoms, parenting behavior, and their children’s internalizing and externalizing behavior.

**Results:**

Results showed that mothers with higher ACE scores reported more maternal psychopathology symptoms and more internalizing behavior in their children. The mother’s psychopathology in and of itself mediated the link between her ACEs and her child’s internalizing and externalizing behavior. Moreover, an indirect sequential path emerged linking ACEs with the mother’s psychopathology symptoms, which, in return, were linked with hostile parenting. Hostile parenting, in turn, was linked with children’s internalizing and externalizing behavior.

**Discussion:**

These findings highlight the complicated and intertwined ways in which adverse experiences early in the mother’s life might put her child’s wellbeing at risk. The findings suggest that ACEs are linked to maternal affect dysregulation, which interferes with parenting, increasing the risk of behavior problems in children. The findings underscore the need to assess mothers’ adverse history, psychological distress, and parenting behavior, and provide treatments that can reduce the intergenerational transmission of early adverse experiences.

## Introduction

Exposure to adversity in early childhood can reverberate across life and jeopardize one’s health and well-being. Adverse childhood experiences (ACEs) refer to childhood abuse, neglect, and household dysfunction occurring prior to age 18 (1). Research has shown trajectories from ACEs to one’s health problems (2, 3), psychopathology (4, 5), and socioemotional obstacles (6). ACEs not only have long-term effects on those who were exposed to early traumas but are also linked with intergenerational risk. Studies from the developmental and family fields have documented links between caregivers’ ACEs and their children’s development and adaptation from birth to adolescence [e.g., (7, 8)]. For example, cumulative ACEs in parents predicted their children’s depression and/or anxiety (9) and ADHD (10). Mothers’ ACEs predicted their children’s anxiety (11), depression (12), chronic stress (13), and externalizing and internalizing behavior (14–17).

However, the hypothesized mechanisms for explaining the intergenerational link between parents’ ACEs and their children’s behavior problems are still to be explored (17–19). We proposed and examined the mediating links between mothers’ ACEs and their children’s behavioral problems through two mediators: the mothers’ psychopathology and the mothers’ parenting.

### Mothers’ ACEs, their psychopathology, and their children’s behavior problems

Exposure to early adversities during childhood is a well-documented risk factor for adults’ mental health. Since the pioneering study of Felitti et al. (20), a host of studies have demonstrated the association between ACE scores and depression (21), anxiety (22), PTSD (23), alcohol dependence (24), substance abuse (25), somatoform dissociation (26), dissociative symptoms (27), and suicide attempts (28). Moreover, the effect of ACEs on mental health is cumulative, meaning that exposure to a greater number of ACEs increases the risk of later mental health problems (3). Research from the parental literature that focused on maternal ACEs similarly found that mothers’ ACEs predicted their depression, anxiety, PTSD (6, 29, 30), and suicidal ideation (31).

Several explanations have been proposed for the association between ACEs and later psychopathology. Explanations have focused on the hyper-reactivity of the Hypothalamic-Pituitary-Adrenal (HPA) axis (32), dysregulation of the immune system (33), and psychological (34), and psychosocial (35) factors. Hoppen and Chalder (4) proposed a *trans*-diagnostic biopsychosocial r4’isk model in which exposure to early ACEs leads to the dysregulation of the autonomic nervous system and the hyper-reactivity of the HPA axis, which in turn, interfere with the stress response and immune system, increasing one’s risk for physical and emotional diseases. Given that these disturbances occur when the brain is developing, they interfere with developmental processes, creating socioemotional difficulties that lead to impaired social and psychological functioning during adulthood (4).

We conducted our study during the early stages of the COVID-19 pandemic. The outbreak of COVID-19 has led to a worldwide increase in mental health problems [e.g., (36–38)], including in Israel (39). A particularly vulnerable group are mothers of young children, who exhibited the highest level of depressive and anxiety symptoms (40). Correspondingly, studies following the pandemic’s outbreak documented an association between more ACEs and more depressive symptoms (41, 42), anxiety (43), and PTSD (44), especially among mothers (45). Taken together, these findings support the contention that early childhood adversity has a lasting negative effect on the mother’s stress response system, making her more vulnerable to emotional dysregulation during stressful times such as COVID-19.

Ample evidence has established mothers’ psychopathology as a key risk factor for their children’s development and adjustment problems [e.g., (46, 47)]. For example, mothers’ depression (48, 49), anxiety (50), and PTSD (51) increase the risk of children’s behavior problems. Moreover, the mother’s psychopathology symptoms may provide an additional explanation for the link between her early traumas and her child’s well-being [e.g., (52–54)]. Support for this argument comes from research demonstrating that maternal anxiety and depression mediate the link between maternal ACEs and children’s internalizing and externalizing problems (11, 52, 55–57). In another study from Japan mothers’ global psychological distress mediated the association between maternal ACEs and ch7’6’ildren’s mental health (12). Note that the impact of early trauma on later mental health is characterized by a broad range of symptoms rather than a unique, specific mental disorder (4). Therefore, in the current study we considered the mother’s global measure of psychopathology symptoms as a possible mediator of the link between her ACEs and her child’s internalizing and externalizing behaviors.

### Mothers’ ACEs and parenting, and children’s behavior problem

Early trauma may not only predict later psychopathology but could also disrupt the parental caregiving system in adulthood. In line with this argument, studies have reported that women who experienced maltreatment in childhood tended to engage in harsh parenting (58), and were more hostile (59, 60), disengaged and intrusive (60). Mothers with many ACEs also demonstrated more aggression toward their child (61). Furthermore, an adverse history of neglect and abuse increased the risk of the mother engaging in the physical abuse, psychological abuse, and neglect of her own children (62). These children may be at greater risk of experiencing ACEs (52, 63).

Research has also established that mothers who endured ACEs as children tend, as a group, to be less engaged in positive parenting behaviors (64). They exhibit less competence in parenting (59), less sensitivity (60), and less maternal self-efficacy (65), and are less available emotionally to their children (66).

Attachment theory provides a theoretical framework for the association between9’ a mother’s ACEs and her parenting. According to the attachment perspective (67), an infant who was exposed to or experienced continued maltreatment or relational trauma is likely to develop an insecure attachment style. Empirical evidence has shown links between mothers’ ACEs and the rate of insecure attachment styles in adulthood (55, 68), as well as higher rates of unresolved states of mind with regard to attachment, another aspect of insecure adult attachment (69). Insecure maternal attachment style is linked to negative parenting behavior (70). Thus, ACEs increase the risk of an insecure attachment style in the parent, which may lead to negative and insensitive parenting.

Another explanation for the association between a mother’s ACEs and her parenting is that parenting a young child may ignite painful memories in the mother about her early trauma, which may evoke affect dysregulation when faced with common, yet stressful, parenting challenges (53, 55, 71). If the mother has an insecure attachment style, her ability to regulate affective arousal effectively is likely to be compromised (72). Such a mother will also have a hard time separating her past threatening experiences from her current parenting challenges (73), activating her self-preservation instinct. This instinct, in return, may overshadow her parental caregiving system and interfere with sensitive and empathic parenting (74).

Parenting practices and behaviors, both positive and negative, are crucial in predicting children’s socioemotional adjustment [e.g., (75)]. Specifically, a large body of research has indicated a positive link between hostile, harsh maternal parenting and children’s and adolescents’ behavioral and emotional problems (76–78). In contrast, positive parenting strategies, specifically, supportive parenting and maternal warmth, are linked to fewer externalizing and internalizing behaviors (79).

Given that parenting behavior is a key factor in children’s behavior and is associated with ACEs, parenting behavior may be another mechanism accounting for the link between the mother’s ACEs and her child’s behavior problems (16). However, studies investigating this possibility have yielded inconsistent findings. On one hand, maladaptive and less responsive parenting such as shouting, slapping, and hostility mediated the association between maternal ACEs and children’s internalizing and externalizing difficulties (80, 81). On the other hand, harsh parenting such as corporal punishment and maternal sensitivity did not mediate the association between maternal ACEs and children’s internalizing and externalizing difficulties (82, 83). Therefore, more research is needed to clarify the mediating role of parenting in the link between maternal ACEs and children’s internalizing and externalizing difficulties.

### The current study

In line with the efforts to identify the mediating mechanisms linking mothers’ ACEs with their children’s socioemotional adaptation, we proposed and tested two sequentially mediating links between maternal ACEs and children’s internalizing and externalizing behaviors *via* two mediators. The first mediator–the mother’s psychopathology symptoms–is based on previous findings associating ACEs with adults’ mental and psychological difficulties. Thus, we posited that the mother’s psychopathology symptoms may create a toxic environment for her child and increase the risk of the child’s developing internalizing and externalizing behavior. The second mediator–the mother’s parenting behavior–is grounded in attachment theory’s claim that the mother’s exposure to trauma at a young age may result in her having an insecure attachment style which may lead to more negative and less positive parenting practices.

**FIGURE 1:**
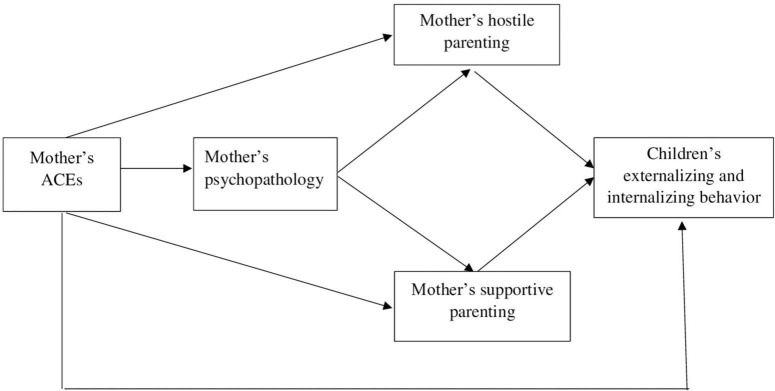
Theoretical mediation model for the association between the mother’s ACEs and her child’s behavior *via* the mother’s psychopathology and her hostile or supportive parenting.

Moreover, parental psychopathology and parenting are also interconnected. Mounting evidence from perinatal research points to maternal psychopathology as preceding parenting behaviors [e.g., (84–86)]. Therefore, we propose that a mother with a history of adverse experiences during her childhood is more likely to suffer from psychopathology symptoms. These symptoms may lead to more hostile parenting and less supportive parenting, which in turn predict her child’s internalizing and externalizing behaviors. Figure 1 presents our conceptual model.

Hence, we hypothesized that:

(1) The mother’s ACEs will be associated with higher rates of maternal psychopathology, more hostile parenting, less supportive parenting, and more internalizing and externalizing behaviors in her children.
(2) The mother’s psychopathology and hostile and supportive parenting will mediate the links between her ACEs and her children’s internalizing (H2a) and externalizing (H2b) behavior. Specifically, a mother with higher ACEs score will have more psychopathology symptoms. These symptoms will lead to both more hostile parenting and less supportive parenting, which, in turn, will lead to more internalizing and externalizing behavior in the child.

## Materials and methods

### Participants

Two hundred and sixty-two Israeli mothers of children between the ages of 3 and 12 years old volunteered for the study. Of the 262, 153 mothers completed all of the study’s questionnaires and comprised the final sample. We used the respondents’ IP address to ensure that no duplicate cases were included in our sample. Independent sample *t*-tests and chi-squared tests found no significant differences between the main demographic variables such as age, education, income and household composition of those who completed the questionnaires entirely and those who did not. The children ranged in age from 3 to 12.8 years old (M = 7.06, SD = 2.54). Girls comprised 52% of the sample. The majority of the children (56.9%) were firstborn, healthy prior to COVID-19 (95.4%) and all attended public schools. The mothers’ mean age was 38.96 years old (SDage = 5.82). Most of them (94.7%) were cohabiting with a spouse and 57.5% of them had a post-secondary education. Most mothers (90.9%) indicated that they were in good or very good physical health before COVID-19, and none reported being sick with COVID-19 prior to or during the time of the study. The majority of mothers indicated above average (56.9%) and average (40%) pre-COVID-19 family incomes according to Israeli standards.

### Measures

We used several established methods to test the factors we proposed.

#### Mother’s adverse childhood experiences

The Adverse Childhood Experiences Questionnaire [ACE; (1)] was utilized. The ACE consists of 10 yes/no questions related to the respondents’ first 18 years of life. The questionnaire items are categorized into three main groups: abuse (including emotional, physical, and sexual abuse), neglect (including emotional and physical neglect), and household dysfunction (including substance use, mental illness, parental separation or divorce, mother being treated violently, and having an incarcerated household member). “Yes” answers receive 1 point and “No” answers receive 0 points, yielding a total score ranging from 0 to 10. Higher scores reflect more adverse experiences. In the current sample, 43.3% of the mothers reported zero ACE, 19.1% of the mothers reported one ACE, 13.8% reported two ACEs, 8.6% reported three ACEs and 15.2% reported four ACEs or more. Cronbach’s alpha for this sample was 0.72.

#### Mothers’ psychopathology symptoms

We used the Global Severity Index (GSI) of the Brief Symptom Index [BSI; (87)] questionnaire to assess the mothers’ reported psychopathology. The BSI is comprised of 53 items that measure psychopathology symptoms on a 5-point Likert-type scale, ranging from 0 (*not at all*) to 4 (*extremely*). Respondents were asked to answer questions such as: “Over the last month, to what extent did you feel no interest in things?” The GSI evaluates the combination of the number and severity of the psychopathology symptoms, with higher scores reflecting greater psychopathology. The BSI is a widely used, psychometrically validated, reliable questionnaire of adult psychopathology (88). Internal consistency of the GSI in the current study was very good (Cronbach’s alpha = 0.94).

#### Mothers’ parenting behavior

The Parent Behavior Inventory [PBI, (89)] was used to evaluate the mothers’ parenting behavior. The PBI comprises 20 items, rated on a 6-point Likert scale from 0 (*not at all* or *never*) to 5 (*always*) that produces two independent scales: supportive/engaged and hostile/coercive. Each of these scales contains 10 items (e.g., “I have pleasant conversations with my child”; “I say mean things to my child that makes him/her feel bad”). The scale was designed for and has been used with parents of pre-school-age and school-age children (13, 89). In the current study the two scales had adequate internal consistency (Cronbach’s alpha = 0.78 and 0.81 for the hostile/coercive and the supportive/engaged scales, respectively).

#### Children’s behavior problems

We used the Child Behavior Checklist questionnaire to measure this outcome variable. This questionnaire has two versions, depending on the child’s age. For children 3–5, mothers completed the CBCL 11/5- 5-years-old (90), which contains 99 items, such as “can’t sit still, is restless, or hyperactive.” For children six and older, mothers completed the CBCL 6–18 years old (91), which contains 113 items, such as “not liked by other kids.” In both versions, items are presented on a 3-point Likert-type scale, ranging from 0 (*not true*), through 1 (*somewhat or sometimes true*), to 2 (*very or often true*). The two versions yield two identical clusters: internalizing behavior and externalizing behavior. In our study, both clusters demonstrated good internal consistency (for younger children: internalizing behavior: Cronbach’s alpha = 0.88; externalizing behavior: Cronbach’s alpha = 0.90; for older children: internalizing behavior: Cronbach’s alpha = 0.83, externalizing behavior: Cronbach’s alpha = 0.91). The CBCL has standardized scores with Israeli norms for each age category (92). There were no significant correlations between the children’s age and the raw scores of their internalizing or externalizing behavior in any age group. Thus, we converted the raw scores into *t*-scores for each age group and used them across the two age groups.

### Procedure

Mothers were recruited through social networks for parents (such as parents’ WhatsApp groups and Facebook) and through the snowball technique shortly after the outbreak of COVID-19 in Israel (from mid-March until the end of April 2020). The recruitment message described the study’s aim in general terms as the desire to learn about parenting and children, specified the required age of the children and provided a link to the online survey. The data were collected online by an open survey (*via* Qualtrics). The first page of the survey described the study’s aims, mentioned the investigators’ names and affiliations, and indicated the estimated length of time needed to complete the survey. The mothers were then invited to sign an informed consent. Participation was anonymous and voluntary. In addition, the mothers were informed that they could end their participation whenever they wanted to do so. There was no reward or compensation for participation. The study was approved by our institution’s Ethics Committee (ref. 2020079). Mothers were asked to consider only one of their children in the specified age range when completing the questionnaires. Based on the child’s age, they were branched to the relevant CBCL form. Only the research’s team could enter the survey by a password. The study’s questionnaires were randomized to reduce bias. Participants were not obligated to answer all of the items. However, at the end of each questionnaire a message notifying the participants about missing items appeared. Participants could go back and fill in the missing items or change their answers before proceeding to the next questionnaire.

### Data analysis

Prior to testing the study’s hypotheses, we used Pearson’s correlations and *t*-tests to conduct preliminary analyses of the associations between various demographic factors and the study’s variables to identify possible confounders. To test H1 we ran Pearson’s correlations for the associations between the mothers’ ACEs, their psychopathology, and parenting, and the children’s internalizing and externalizing behavior. To test H2 we used Hayes (93) PROCESS model 81 with 5,000 bias-corrected bootstrap samples. We conducted the analyses separately for the child’s internalizing and externalizing behaviors, resulting in two models. In each model we tested the direct and indirect links between the mother’s ACEs (X) and the child’s behaviors (externalizing or internalizing; Y) with the mothers’ psychopathology symptoms as the first mediator (M1), followed by their hostile or supportive parenting as sequential parallel mediators (M2, M3, respectively). We considered the effects significant at *p* < 0.05. When the 95% confidence interval included 0, we inferred a significant indirect effect at the 0.05 level. All analyses were done using IBM SPSS Statistics version 27 and the PROCESS macro version 3.5 for SPSS (93).

## Results

Preliminary analyses of the associations between the demographic variables and the study’s variables revealed that the child’s age was significantly and negatively associated with the mothers’ reports of supportive parenting (*r* = −0.17, *p* = 0.03). Thus, mothers of older children reported fewer supportive and engaged parenting behaviors. The child’s gender was significantly associated with the mother’s GSI [F (151, 1) = 4.47, *p* = 0.04] such that mothers of boys reported more psychopathology symptoms than mothers of girls. The child’s gender was also associated with the mothers’ report of their children’s internalization behavior [F (145, 1) = 5.33, *p* = 0.02] indicating that girls had more internalizing behavior than boys. Thus, we included the child’s age and gender as covariates in further analyses.

To test H1, we ran Pearson’s correlations to examine the associations between the mothers’ ACEs, their psychopathology symptoms, supportive parenting, and hostile parenting, and their children’s internalizing and externalizing behaviors. Table 1 presents the descriptive information (means and standard deviations or counts and percentages, as appropriate) and correlations among the study’s variables.

**TABLE 1:** Descriptive statistics of the study’s variables and their intercorrelations (*N* = 153).

	M/SUM	SD	Mother’s ACEs	Mother’s GSI	Mother’s supportive parenting	Mother’s hostile parenting	Child’s externalizing behavior
Mother’s ACEs	1.48	1.82					
Mother’s GSI	0.58	0.38	0.30***				
Mother’s supportive parenting	4.42	0.50	0.03	–0.02			
Mother’s hostile parenting	1.29	0.59	0.07	0.36**	–0.14		
Child’s externalizing behavior	50.02	9.58	0.13	0.37**	−0.18*	0.49**	
Child’s internalizing behavior	51.06	10.20	0.22**	0.45**	−0.16*	0.37**	0.60**

ACE, adverse childhoods experience; GSI, psychological symptoms.

*p < 0.05, **p < 0.01, ***p < 0.001.

As predicted, there were positive and significant correlations between the mothers’ ACE scores and their reports of their psychopathology, and their children’s internalizing behavior. Mothers who experienced more early traumas reported more psychopathology symptoms and more internalizing problems in their children. However, contrary to our hypothesis the mothers’ ACEs were not correlated with their children’s externalizing behavior or with the mothers’ reports of their hostile or supportive parenting. In addition, there were significant positive correlations between the mothers’ psychopathology and their hostile parenting and their reports of their children’s internalizing and externalizing behavior. Moreover, the mother’s parenting behaviors, be it supportive or hostile, was correlated with the child’s behavior, both internalizing and externalizing. In sum, H1 was partially confirmed.

**FIGURE 2:**
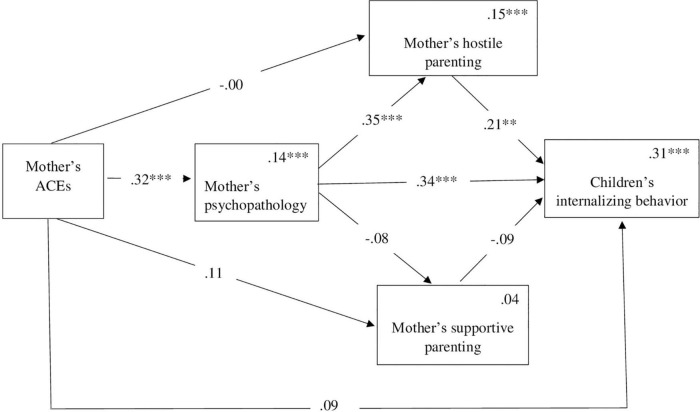
Mediation model for the association between the mother’s ACEs internalizing behavior *via* the mother’s psychopathology and her hostile or supportive parenting. Numbers above the lines are standardized coefficients. Numbers in the upper right-hand corner in the rectangles present R^2^. While not presented in the figure, the child’s age and gender were included as covariates in the model. **p < 0.01, ***p < 0.001.

To test H2 regarding the mediated links between the mother’s ACE and her children’s behavior *via* the mother’s psychopathology symptoms and her supportive or hostile parenting, we used two models: one for internalizing behavior (H2a) and one for externalizing behavior (H2b). The mediation model predicting a child’s internalizing behavior from the mother’s ACEs *via* the mother’s psychopathology symptoms and her hostile or supportive parenting was significant [F (6, 137) = 10.11, *p* < 0.001], accounting for 31% of the variance. Figure 2 depicts the links between the model’s variables. While not presented in the figure for the sake of visual clarity, the child’s age and gender were included in the models as covariates. No direct effect was found between the mother’s ACEs and the child’s internalizing behavior. Table 2 presents the indirect effects. As the table indicates, the indirect link between the mother’s ACEs and her children’s internalizing behavior *via* the mother’s psychopathology was significant. Moreover, as predicted, the link between the mothers’ ACEs and the children’s internalizing behavior was mediated sequentially *via* the mothers’ psychopathology symptoms and hostile parenting. The higher the mother scored on having experienced ACEs, the more severe her psychopathology was. This psychopathology was linked to more hostile parenting, which was linked to more internalizing behavior among the children. However, the indirect paths between the mother’s ACEs and the children’s internalizing behavior *via* the mother’s parenting behavior, hostile or supportive, each on its own, were not significant. Finally, the indirect sequential path through the mother’s psychopathology and her supportive parenting was not significant.

**TABLE 2:** Indirect paths between mother’s adverse childhood experiences (ACEs) and child’s behavior *via* mother’s psychopathology and parenting.

	Effect	SE	95% CI
**Child’s internalizing behavior:**			
Mother’s ACEs → mother’s psychopathology → internalizing behavior	**0.11**	**0.05**	**[0.03, 0.22]**
Mother’s ACEs → mother’s hostile parenting → internalizing behavior	–0.00	0.02	[−0.04, 0.04]
Mother’s ACEs → mother’s supportive parenting → internalizing behavior	–0.01	0.00	[−0.03, 0.00]
Mother’s ACEs → mother’s psychopathology → mother’s hostile parenting → internalizing behavior	**0.02**	**0.01**	**[0.00, 0.05]**
Mother’s ACEs → mother’s psychopathology → mother’s supportive parenting → internalizing behavior	0.00	0.00	[−0.00, 0.01]
**Child’s externalizing behavior:**			
Mother’s ACEs → mother’s psychopathology → externalizing behavior	**0.07**	**0.04**	**[0.00, 0.16]**
Mother’s ACEs → mother’s hostile parenting → externalizing behavior	0.00	0.03	[−0.07, 0.07]
Mother’s ACEs → mother’s supportive parenting → externalizing behavior	0.01	0.01	[−0.04, 0.005]
Mother’s ACEs → mother’s psychopathology → mother’s hostile parenting → externalizing behavior	**0.04**	**0.02**	**[0.01, 0.09]**
Mother’s ACEs → mother’s psychopathology → mother’s supportive parenting → externalizing behavior	0.00	0.00	[−0.00, 0.01]

Significant paths marked in bold.

**FIGURE 3:**
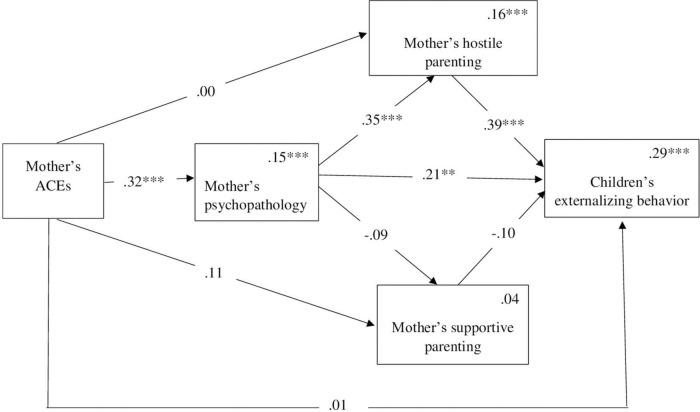
Mediation model for the association between the mother’s ACEs externalizing behavior *via* the mother’s psychopathology and her hostile or supportive parenting. Numbers above the lines are standardized coefficients. Numbers in the upper right-hand corner in the rectangles present R^2^. While not presented in the figure, the child’s age and gender were included as covariates in the model. **p < 0.01, ***p < 0.001.

The model predicting the child’s externalizing behavior from the mother’s ACEs *via* her psychopathology symptoms and her hostile and supportive parenting was also significant [F (6, 138) = 9.64, *p* < 0.001] and accounted for 29% of the variance. As Table 2 indicates, the pattern of the indirect paths was similar to those for internalizing behavior. No direct effect was found between the mother’s ACEs and the child’s externalizing behavior. The indirect link between the mother’s ACEs and the children’s externalizing behavior *via* the mother’s psychopathology symptoms was significant. Furthermore, as predicted, the link between the mothers’ ACEs and the children’s externalizing behavior was mediated sequentially *via* the mothers’ psychopathology and hostile parenting. Nevertheless, the indirect sequential path through the mothers’ psychopathology symptoms and supportive parenting was not significant. Thus, the higher the mother scored on having experienced ACEs, the more severe her psychopathology was. This psychopathology was linked to more hostile parenting, which was linked to more externalizing behavior among the children. The indirect links between the mother’s ACEs and her children’s externalizing behavior *via* the mother’s hostile or supportive parenting, on their own, were not significant. Figure 3 presents the direct and indirect links between the model’s variables. While not presented in the figure for the sake of visual clarity, the child’s age and gender were included in the models as covariates.

## Discussion

The study’s aim was to explore the potential mediating mechanisms accounting for the intergenerational association between a mother’s early adverse experiences and her child’s behavior problems. We focused on the mother’s psychopathology and her hostile or supportive parenting behavior as mediators of this association. In support of H1 we found that mothers with higher ACE scores reported more psychopathology symptoms and more internalizing behavior in their children. However, mothers’ ACEs were not associated with their reports of their parenting behaviors style, either supportive or hostile, or with the children’s externalizing behaviors. Supporting H2, we found two indirect paths between the mother’s ACEs and the child’s behavior problems. The first indirect path was *via* the mother’s psychopathology. Specifically, higher ACE scores led to more psychopathology symptoms and distress, which, in turn, predicted more internalizing and externalizing behavior in the children. The second indirect path was a sequential mediation *via* the mother’s psychopathology and hostile parenting. The more ACEs the mother had as a child, the more psychological distress she reported as an adult. The mother’s psychological distress led to hostile parenting, which, in turn, led to more internalizing and externalizing behavior in her child. Interestingly, neither of the parenting behaviors on their own, supportive or hostile, mediated the association between the mother’s ACEs and the child’s internalizing and externalizing behavior. In addition, we found no sequential mediation *via* the mother’s psychopathology symptoms and supportive parenting on the association between the mother’s ACEs and the child’s internalizing and externalizing behavior.

The correlation between the mother’s ACEs and her psychopathology symptoms that we found joins substantial empirical evidence of the association between ACEs and later psychopathology (2, 3, 6, 22, 53, 94), particularly following the outbreak of COVID-19 (41, 42, 44, 45). This result suggests that experiencing abuse, neglect, and household dysfunction prior to age 18 increases the risk of later emotional difficulties for mothers, particularly during stressful times such as the outbreak of COVID-19. Notably, in the current study the association between the mother’s adverse experiences and her later distress emerged even in a low-risk sample, highlighting the profound, devastating, and long-lasting implications of early adverse circumstances even in relatively robust groups. To conclude, our findings, as well as other evidence, support current *trans*-diagnostic bio-socio-emotional understandings that childhood adversity interferes with the mother’s ability to self-regulate and deal with stress, increasing her risk for psychopathology (4).

The association between the mother’s ACEs and her child’s internalizing behavior resonates with previous findings about the intergenerational transmission of trauma (11, 17, 55). It confirms that children of mothers with adverse childhood experiences are at heightened risk for behavior problems. Interestingly, and contrary to previous studies, we did not find a simple correlation between the mothers’ ACEs and the child’s externalizing behavior. We suggest that this particular finding may reflect the special times of COVID-19 and its ramifications for children’s and adults’ mental health. It is possible that the pandemic-related rise in children’s fears, worries, anxiety, and depression (95–98) led mothers to report more internalizing behavior. Furthermore, it is possible that mothers with more ACEs were more distressed by the pandemic and may have projected their own fears, concerns, and negative affect onto their children, resulting in more reports of internalizing behaviors. These mothers may have also unconsciously reinforced their children’s internalizing difficulties by overanalyzing their behavior, looking for signs of distress and being overprotective of them (18). Importantly, although the simple correlation between the mother’s ACEs and her child’s externalizing behavior was not significant, the indirect links *via* the mother’s psychopathology symptoms and hostile parenting were significant, as we will discuss later.

The mothers’ psychopathology played a key role in mediating the association between their history of trauma and the children’s behavior. This finding accords with a growing number of studies demonstrating that the mother’s psychopathology creates a path through which her early adverse experiences impact her child (57, 99, 100). Moreover, whereas the majority of previous studies focused on young children from Western countries, our study included older children and was conducted in Israel, which is unique in its cultural composition, consisting of Westernized and non-Westernized cultural groups. Thus, our findings support and expand previous research indicating that the mother’s psychological distress is an important factor accounting for the intergenerational transmission of the adverse events she experienced during her childhood to her child.

Importantly, and as hypothesized, we found that the mother’s psychopathology symptoms not only mediated the association between her ACEs and the child’s internalizing and externalizing behaviors, but also was involved in sequential mediation with the mother’s hostile parenting. Thus, when a mother experienced more trauma as a child, she was likely to experience more distress and report more mental difficulties. These outcomes were linked with harsher, more hostile and agitated parenting, which predicted more reports about the problematic behavior of her child. Note that the mother’s hostile parenting on its own did not mediate the link between her ACEs and her child’s behavior problems. This finding highlights the importance of the mother’s psychopathology as a leading factor that can put both parenting and the child’s well-being at risk (101). In line with previous research embedded within attachment theory [see Cooke et al. and Verhage et al. (55, 102) for a review], we argue that ACEs may evoke painful attachment-related memories of trauma, loss, and unfavorable care, all contributing to the increased risk of the mother’s developing an insecure attachment style. Insecure attachment and unprocessed traumatic memories may lead to affect dysregulation, which, in turn, may interfere with the mother’s ability to provide sensitive parenting. Insensitive parenting may lead to the mother’s limited ability to regulate her child’s emotions, putting the child’s development and socioemotional adjustment at risk (103).

The sequential mediation through the mother’s psychopathology symptoms and hostile parenting we documented varies from the results of a recent longitudinal study by Shih et al. (19) that examined sequential mediation of the link between maternal ACEs and children’s internalizing behaviors at ages 4–6 *via* maternal anxiety and positive parenting. Shih and her colleagues reported that the mother’s anxiety and positive parenting mediated the link between the mother’s adversity and the child’s internalizing behavior separately, not sequentially. The differences in the results could be due, in part, to differences in the children’s ages and the different parenting practices that were measured. Nevertheless, our innovative finding needs further replication.

Interestingly, supportive and caring parenting, although associated with the children’s internalizing and externalizing behavior, did not mediate the association between the mother’s ACEs and the child’s internalizing and externalizing behavior on their own, or sequentially with the mother’s psychopathology symptoms. These findings echo previous results that found no associations between the mother’s ACEs and positive parenting practices (71) or sensitive and scaffolding parenting (104). However, it contradicts previous findings demonstrating that positive parenting (i.e., the fostering of cognitive growth) mediated the link between the mothers’ ACEs and their pre-schoolers’ internalizing behavior (19). (71) argued that their failure to find an association between ACEs and positive parenting reflected a social desirability bias and the over-reporting of positive parenting practices, which may be the case in our study as well. Alternatively, the lack of association may also be linked with the newly emerging empirical and clinical data regarding the suppressing effect of early childhood adversity on the dopamine system and the parental reward system, which may lead to the inability to enjoy and report positive parenting practices (74).

### Contributions, limitations, and future directions

The current study joins and expands the literature on the possible pathways through which mothers’ ACEs might be a risk for their children’s behavior. Its main added contribution lies in identifying the sequential mediation of the link between the mother’s ACEs and the child’s internalizing and externalizing behavior first by the mother’s psychopathology and then by her hostile parenting. It highlights the complicated ways in which adversity is transferred from caregiver to offspring. Furthermore, this study emphasizes the mother’s psychological distress as a crucial factor in the association between her history of being hurt and her child’s well-being, mediated on its own, and also interfering with the mother’s parenting.

Nevertheless, these contributions need to be viewed in light of some limitations. First, although the ACE questionnaire is considered to be a reliable measure of childhood adversity (105), it is based on retrospective reports and prone to recall bias. Second, all of the study’s measures were based on the mothers’ self-reports. Thus, there is a risk of shared method variance as well as desirability bias. Future studies can benefit from using multiple informants including fathers, teachers, and the children themselves. Third, our sample was relatively modest in size and homogeneous in terms of the families’ SES, the mothers’ educational level, and two-parent households. In addition, we used a convenience sample of volunteers who felt comfortable using social media and were willing to complete an online survey. Moreover, the data were collected during the COVID-19 pandemic, which affected the socioemotional adaptation and functioning of the parents and children. These characteristics of our sample limit our ability to generalize the findings. They also make it difficult for us to draw broad-based conclusions about the intergenerational effects of the mothers’ ACEs on other family contexts among less advantaged populations and during less stressful times. Fourth, the study included only mothers. Additional studies that examine the role of fathers’ ACEs on their children’s behavior are clearly needed. Finally, the data were collected concurrently, precluding causal, directional, and reciprocal inferences. While our underlying assumption was that maternal psychopathology leads to negative parenting that is hostile and less supportive, and behavior problems in children, it is equally possible that the opposite is true: children’s difficult behavior leads to maternal psychopathology and negative parenting. Longitudinal designs with larger and more heterogeneous samples are needed to test alternative models and further understand the mechanisms that explain the association between the caregiver’s early adverse experiences and their child’s difficulties later in life. In addition, longitudinal designs that follow children who have been exposed to ACEs into adulthood and parenthood can help us better understand the developmental trajectory associated with ACEs.

### Clinical implications

Given the increased risk documented in the literature and found in our study associated with the mother’s adverse childhood experiences, her mental health, and her children’s socioemotional adjustment, healthcare providers for families and children are advised to screen and assess mothers’ (and probably fathers’) past traumatic experiences. Interventions for a parent who experienced adversity or trauma can help the parent better cope with his/her history and can also be protective for the child. For example, trauma informed care that includes validation and recognition of the effects of traumatic events, common coping strategies, and effective treatments (106) can help mothers limit the impact that their negative history has on their children.

Furthermore, treatment interventions for children need to include an assessment of the mother’s early childhood experiences, particularly her ACEs, but also her attachment-related experiences and scripts, which are linked with an increased risk of her psychopathology and, consequently, her parenting. Evidence-based treatment interventions that combine an attachment and trauma lens for young children [e.g., ABC, (107); COS, (108); CPP, (109); GABI, (110)] and adolescents [ABFT, (111)] may serve as guidelines for universal and specialized treatment plans for traumatized mothers and their children.

Finally, it is important to remember that the intergenerational transmission of childhood adversity can be broken (112). Research and clinical work have demonstrated the utility of assessing and utilizing parents’ positive childhood experiences, such as the approach of “Angels in the Nursery” (113, 114), as a source of resilience when working with parents who suffered early trauma. Furthermore, parents’ reflective functioning and mentalization skills have proven effective in breaking the link between parents’ childhood trauma and children’s insecure attachment (115). Thus, interventions such as the Family Cycle (116) or MBT-C (117) that enhance the parents’ reflective functioning and mentalization provide guidelines for intervening and can be used to improve parents’ understanding of their children’s emotional and attachment-related needs.

## Data availability statement

The raw data supporting the conclusions of this article will be made available by the authors, without undue reservation.

## Ethics statement

The studies involving human participants were reviewed and approved by Academic College of Tel Aviv Yaffo’s Ethics Committee approval reference #2020079. The patients/participants provided their written informed consent to participate in this study.

## Author contributions

Both authors listed have made an equally substantial, direct, and intellectual contribution to the work, and approved it for publication.
